# Pinhole-type two-dimensional ultra-small-angle X-ray scattering on the micrometer scale

**DOI:** 10.1107/S1600577513023205

**Published:** 2013-11-02

**Authors:** Hiroyuki Kishimoto, Yuya Shinohara, Yoshio Suzuki, Akihisa Takeuchi, Naoto Yagi, Yoshiyuki Amemiya

**Affiliations:** a Materials Research and Developments HQS, Sumitomo Rubber Industries Ltd, 2-1-1 Tsutsui, Chuo, Kobe, Hyogo 651-0071, Japan; b Department of Advanced Materials Science, The University of Tokyo, 5-1-5 Kashiwanoha, Kashiwa, Chiba 277-8561, Japan; c Research and Utilization Division, Japan Synchrotron Radiation Research Institute, 1-1-1 Kouto, Sayo, Hyogo 679-5198, Japan

**Keywords:** ultra-small-angle X-ray scattering, hierarchical structures, nanocomposites

## Abstract

A pinhole-type two-dimensional ultra-small-angle X-ray scattering set-up at a so-called medium-length beamline at SPring-8 is reported. A long sample-to-detector distance, 160.5 m, can be used at this beamline and a small-angle resolution of 0.25 µm^−1^ was thereby achieved at an X-ray energy of 8 keV.

## Abstract 2

A pinhole-type ultra-small-angle X-ray scattering set-up that enlarges the accessible *q*-range to 0.25 µm^−1^ is described.

## 1. Introduction   

Soft matter generally shows hierarchical structure; the control of such hierarchical structure is important for developing high-performance products. Rubber containing nanoparticles is such a system; the hierarchical structure of the nanoparticles in rubber has a close relationship with reinforced mechanical and viscoelastic properties of filled rubber (reinforcement effect) (Wang, 1998 ▶). For clarifying the mechanism of the reinforcement effect, the structure of nanoparticles needs to be studied over a wide structural scale; hence both small-angle X-ray scattering (SAXS) (Ehrburger-Dolle *et al.*, 2001 ▶; Kishimoto *et al.*, 2008 ▶; Koga *et al.*, 2008 ▶; Chevigny *et al.*, 2011 ▶; Baeza *et al.*, 2012 ▶; Takenaka, 2013 ▶) and X-ray imaging techniques (Kishimoto *et al.*, 2013 ▶) have been used. Apart from the reinforcement effect of nanocomposites, SAXS techniques are widely used to reveal the hierarchical structures of nanoparticle aggregates (Hyeon-Lee *et al.*, 1998 ▶; Kammler *et al.*, 2004 ▶; Rai *et al.*, 2012 ▶). SAXS covers the 1–100 nm size range; this range is not wide enough to cover a large-scale hierarchical structure. To extend the range of SAXS down to lower angles, a scanning method utilizing crystal collimation has been widely used (Bonse & Hart, 1965 ▶, 1966 ▶; Sztucki & Narayanan, 2007 ▶; Ilavsky *et al.*, 2009 ▶). This technique gives excellent ultra-small-angle scattering resolution and can be applied to anisotropic structures by rotating samples even though it is basically a one-dimensional scanning technique. Furthermore, it can be applied to time-resolved studies by reducing the angular range of scanning. Still, for measuring the time evolution of an anisotropic structure, which is often the case with nanoparticle aggregates in rubber, its time resolution is limited due to the time required for rotation of a sample and scanning. High-flux X-rays are required to reduce the limitations, which results in unavoidable radiation damage.

To overcome the difficulty, we have developed a two-dimensional ultra-small-angle X-ray scattering (USAXS) set-up using a long sample-to-detector distance at BL20XU, SPring-8 (Yagi & Inoue, 2003 ▶; Shinohara *et al.*, 2007 ▶). Because a two-dimensional USAXS pattern can be recorded in a single shot, the measurement time is significantly shortened. The amount of X-ray dose is hence reduced, thereby suppressing the radiation damage. The distance between the sample and the USAXS detector is 160.5 m and the X-ray energy used is 23 keV; in this set-up, the small-angle-scattering resolution is limited to *q* > 1.5 µm^−1^ (here, *q* is defined as *q* = 4πsinθ/λ, where 2θ is the scattering angle and λ the X-ray wavelength) which corresponds to a small-angle-scattering resolution of 4.2 µm in real space. There is still a need to measure lower-angle X-ray scattering for clarifying the higher-order structure in a hierarchical structure. In the present study, we used lower-energy X-rays and a smaller beam stop to measure scattering in a much smaller angle. In this paper, we present the details of the USAXS set-up.

## 2. Experimental set-up   

**Figure 1:**
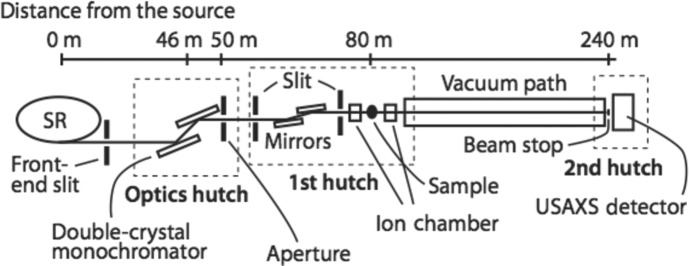
Schematic view of experimental set-up at BL20XU, SPring-8. The distance between the sample and the USAXS detector was 160.5 m.

**Figure 2:**
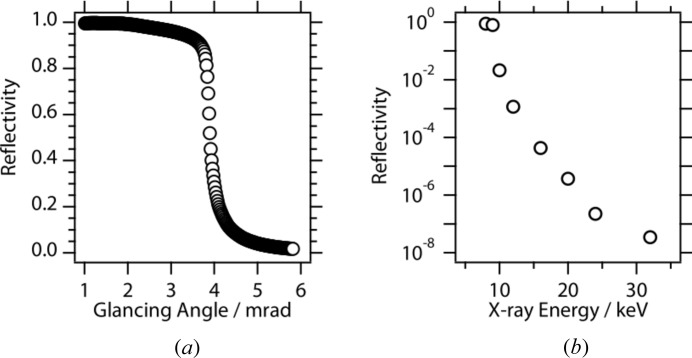
(*a*) Reflectivity using 8 keV X-rays. (*b*) Dependence of two-time reflectivity on X-ray energy at 3.39 mrad.

The USAXS experiments were performed at BL20XU, SPring-8 (Hyogo, Japan) (Suzuki *et al.*, 2004 ▶). A schematic diagram of the experimental set-up is shown in Fig. 1 ▶. The monochromator is a liquid-nitrogen-cooled double-crystal monochromator (Si 111) located at 46 m from the source. The X-ray energy used was 8 keV. The first hutch in the ring building is placed at approximately 80 m from the source, and the second hutch in the Biomedical Imaging Center is at 240 m. The two hutches are connected by a vacuum pipe with an inner diameter of 80 mm. A rectangular aperture (50 µm on a side) was set downstream from the monochromator in the optics hutch. This aperture was used as a virtual light source. A four-quadrant slit was placed at the entrance of the first hutch, and was used to remove parasitic scattering. Two sets of borosilicate glass (Pyrex) mirrors (Sigma Koki, Japan) were used to eliminate higher harmonics. The surface flatness of the mirror was λ/10 at λ = 632.8 nm. The reflectivity of this mirror for 8 keV X-rays is shown in Fig. 2 ▶(*a*). The glancing angle was set to 3.39 mrad, at which the intensity of higher-energy X-rays was reduced as shown in Fig. 2 ▶(*b*); in this way, higher harmonic X-rays such as 24 keV X-rays were removed. Parasitic scattering from the mirrors was removed by using another set of guard slits that was placed just downstream of the mirrors. The specimen was placed just behind the guard slits. There were air gaps of approximately 1 m upstream and downstream of the specimen. In the second hutch, a beam stop was set just downstream of the exit window of the vacuum pipe. The diameter of the beam stop was 2 mm. There was another vacuum pipe of 1 m downstream of the beam stop and an X-ray detector was set just behind the exit window of the pipe. An imaging plate (IP) (Amemiya, 1995 ▶) was used to record two-dimensional USAXS patterns. The use of an IP allows us to record a scattering pattern with a wide dynamic range. The IP was scanned with an off-line scanner (BAS2500, Fuji Film, Tokyo, Japan) with a pitch of 100 µm. The transmittance was recorded using a set of ion chambers. The IP can be replaced with other two-dimensional detectors such as a CCD detector coupled with an X-ray image intensifier (Amemiya *et al.*, 1995 ▶), for performing time-resolved measurements. The distance between the sample and the detector was 160.5 m. The beam size (full width at half-maximum, FWHM) was 0.1 (H) × 0.1 mm (V) at the sample and 0.78 (H) × 0.71 mm (V) at the USAXS detector position, which are comparable with those using 23 keV X-rays.

## 3. Results and discussion   

**Figure 3:**
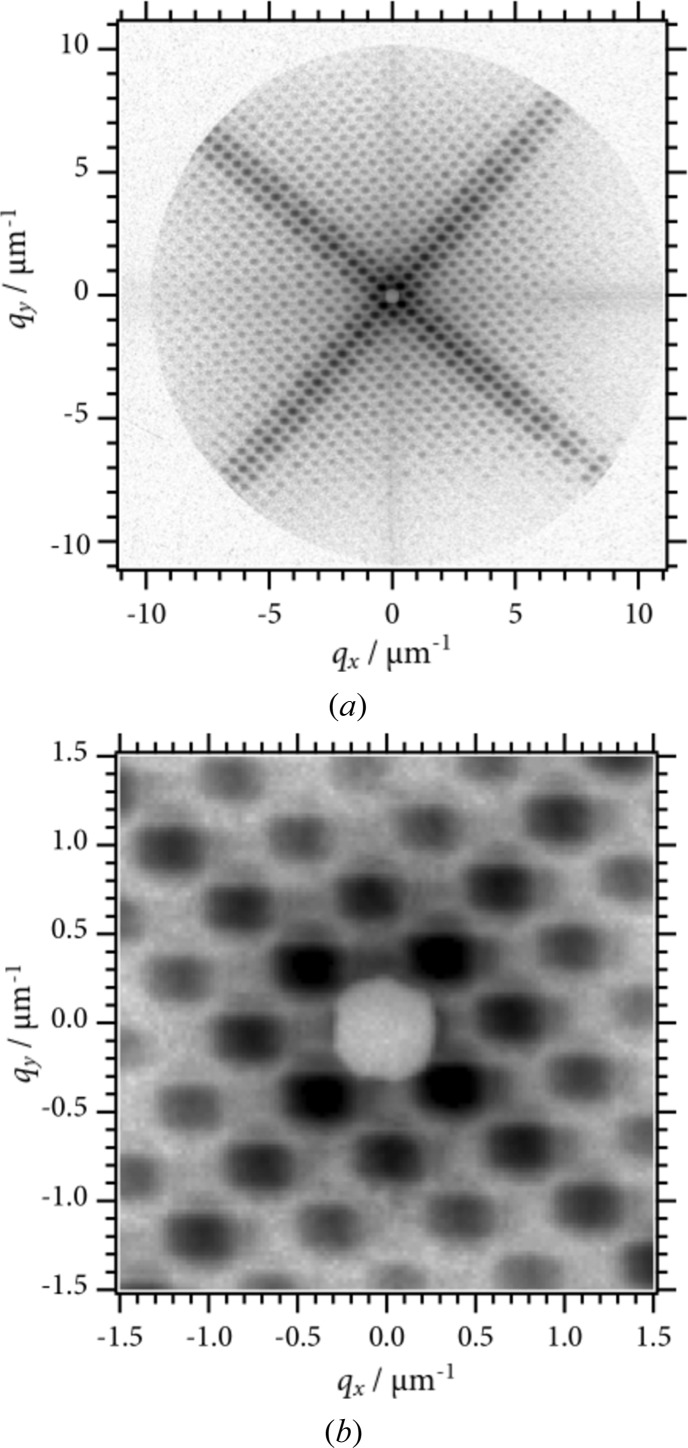
(*a*) USAXS pattern from a copper mesh on a logarithmic scale. (*b*) A blow-up of the central part.

Fig. 3 ▶ shows an USAXS pattern from a copper mesh (G2000HS, Gilder Grids, UK); the pitch, the bar width and the hole width were 12.5, 5 and 7.5 µm, respectively. The image is shown on a logarithmic scale. The exposure time was 500 ms, which was controlled by a shutter installed just downstream of the mirrors. The first-order reflections from the mesh structure were observed around the beam stop and the reflections up to the 21st order were observed. In this image, scattering in the *q*-range of 0.25–10 µm^−1^ is recorded. The crossed pattern originates from the form factor of the rectangular grid hole. The FWHM of each diffraction spot was 0.83 (H) × 0.75 mm (V). There are two factors that broaden the spot size: one is the direct beam size that is determined due to the angular divergence of X-rays and the opening size of the rectangular aperture. As shown in a previous study using 23 keV X-rays, the beam size at the detector position depends on the rectangular aperture size due to the diffraction from the rectangular aperture (Shinohara *et al.*, 2007 ▶). The other factor is spatial coherence length and/or beam size at the sample. The FWHM of the diffraction peak from a periodic structure, Δ*q*, is related to the number of periods within a coherent X-ray beam, *N*, through calculating the FWHM of a Laue function as follows: Δ*q* = 0.88 × 2π/(*Nd*), where *d* is the pitch of the periods. In the present case, the FWHM of the spot is larger than the FWHM of the direct beam, and is divided into the contribution from the size of the direct beam and the broadening due to the width of the Laue function. Based on the assumption that the broadening of a spot is expressed as the root-mean square of the two contributions, the maximum transverse length inside which coherent scattering takes place is estimated to be 0.84 × 10^2^ µm (H) × 1.0 × 10^2^ µm (V). These values are consistent with the transverse coherence length estimated from the size of the rectangular aperture, the X-ray wavelength and the distance between the aperture and the sample: 93 µm. Also, the values are equivalent to the beam size. From the above discussion, we conclude that (i) the beam at the sample is nearly full-coherent, and (ii) a reduction in the beam size at the detector position is required for both minimizing the measurable *q* and Δ*q* values. Further optimization of the mirrors is expected to enhance the performance significantly.

**Figure 4:**
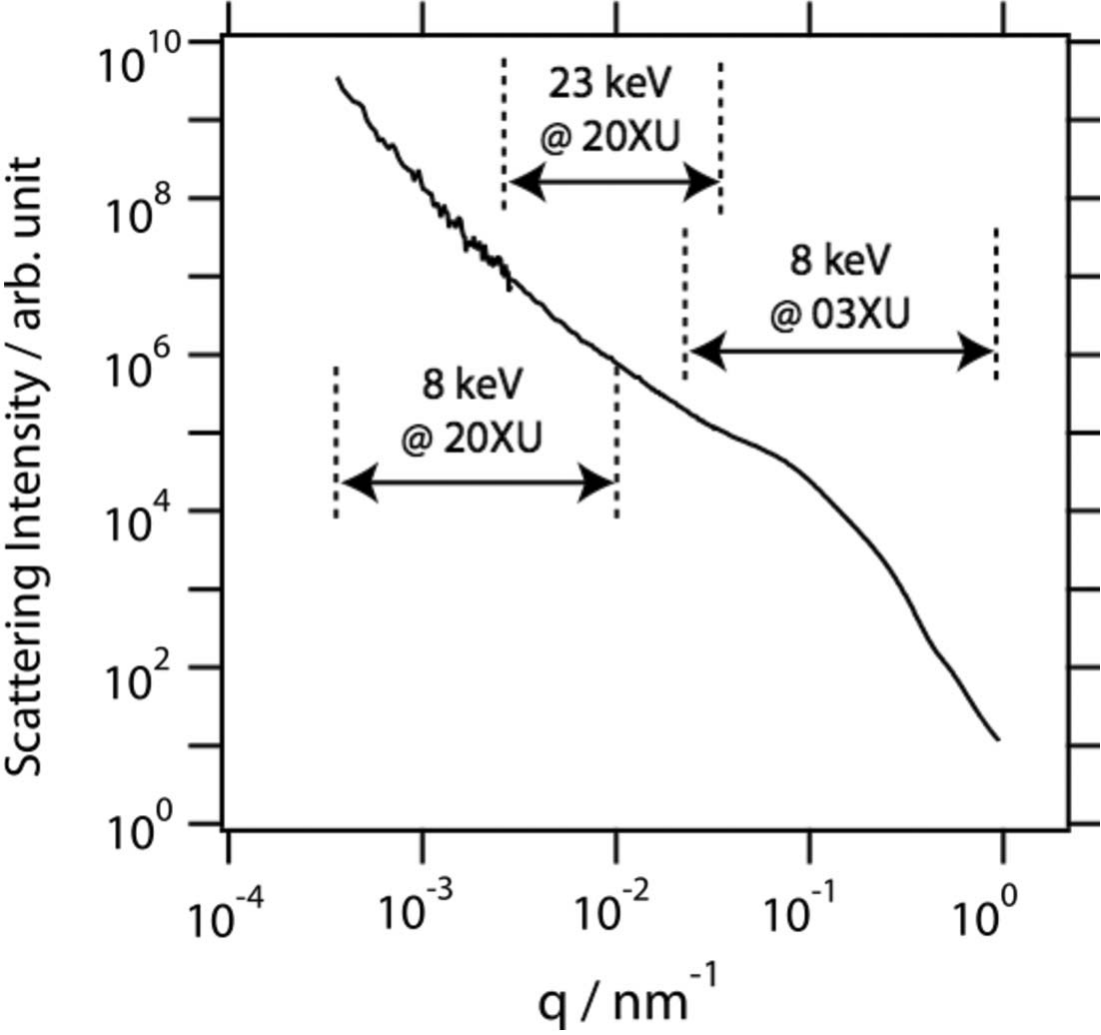
One-dimensional scattering intensity profile of silica particles in styrene–butadiene rubber. The *q*-range covered by different settings is shown.

To elucidate hierarchical structures of filled rubber, scattering over wide *q* should be combined. Fig. 4 ▶ shows a combined one-dimensional scattering intensity profile of styrene–butadiene rubber filled with commercial-grade silica particles (Ultrasil VN3): USAXS using 8 keV X-rays at BL20XU, 23 keV X-rays at BL20XU (Shinohara *et al.*, 2007 ▶), and SAXS using 8 keV X-rays at BL03XU, SPring-8 (Masunaga *et al.*, 2011 ▶) were combined. USAXS and SAXS in the *q*-range 2.5 × 10^−4^–1.0 nm^−1^ was recorded: this corresponds to 6 nm–25 µm in real space. The one-dimensional scattering intensity profiles were obtained by azimuthally averaging two-dimensional data. Detailed structural analysis based on the scattering intensity profile is beyond the scope of the present article; analysis of the anisotropic structural distribution of aggregates/nanoparticles in rubber will also be presented elsewhere. The intensity profiles measured using 8 keV X-rays at BL20XU show significant fluctuation. This fluctuation originates from the speckle observed in this setting owing to the coherence of X-rays (Sutton *et al.*, 1991 ▶). The speckle was observed even in the previous setting, while any intensity fluctuation originating from the speckle was hardly observed in the one-dimensional scattering intensity profiles (Shinohara *et al.*, 2007 ▶). The transverse coherent length, 30 µm, using 23 keV X-rays was smaller than the beam size at the sample position, thereby blurring the speckles and reducing their contrast. The use of lower X-ray energy in the present study increased the coherent length as shown in the previous paragraph and the visibility of the speckle thus increased. The appearance of the speckle leads to the increase of noise, but it opens the possibility of performing X-ray photon correlation spectroscopy (XPCS) (Sutton, 2008 ▶) in this ultra-small-angle region, the details of which will be presented elsewhere. XPCS at this beamline has several advantages over conventional XPCS settings: (i) the limitation on the spatial resolution of detectors is reduced owing to the long sample-to-detector distance; thus, various X-ray area detectors can be used to record speckle pattern; (ii) dynamics that are too fast to measure in conventional XPCS settings can be measured because the time scale slows down when observing at a larger scale at a smaller angle.

The *q*-range in the present setting is comparable with the limiting range of the Rayleigh–Gans (RG) approximation. The applicability of the RG approximation has been discussed greatly for small-angle light scattering (Kerker *et al.*, 1963 ▶; Barber & Wang, 1978 ▶; Zhao & Ma, 2009 ▶), while the applicability of the RG approximation to SAXS has hardly been discussed to our knowledge, except in a few papers (Smith & Dwek, 1998 ▶). Consider the case of spherical particles in a medium. The RG approximation for the differential scattering cross section requires the following two assumptions (van de Hulst, 1957 ▶). Firstly, that the reflection from the surface of the particles is negligible, *i.e.* that δ 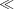 1, where δ is the deviation of the refractive index from unity. This condition is always valid for X-rays in this energy region. The other requirement is that *ka*δ 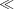 1, where *k* is the wavenumber of the X-ray beam and *a* is the radius of the sphere. In the present case, where δ is of the order of 10^−6^ and *k* is 40.8 nm^−1^, the condition is expressed as *a* 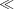 24.5 µm; the RG approximation is, therefore, applicable in the present setting for a sample with a low electron density contrast, which is satisfied for most soft matters. Because most of the studies on filler particles show higher hierarchical structure in this *q*-range, it is of great importance to study the applicability of the RG approximations as in studies of the light scattering of aggregates (Farias *et al.*, 1996 ▶; Chakrabarty *et al.*, 2007 ▶).

In conclusion, a small-angle scattering resolution was extended to *q* = 0.25 µm^−1^. An optimization of the mirrors is expected to enhance the performance significantly. The applicability of the RG approximation needs to be carefully examined. Combination of this two-dimensional USAXS technique and an X-ray imaging technique (Kishimoto *et al.*, 2013 ▶) enables us to elucidate the three-dimensional structural distribution of nanoparticles in rubbery materials, which is a key for improving the design of vehicle tyres.
